# Westminster Hospital

**Published:** 1859-04

**Authors:** 


### *Westminster Hospital.—Epilepsy for Thirty-two years in a Man, aged Forty-four, with Discoloration of the Skin from Nitrate of Silver; Operation of Castration.*

(Under the care of Mr. Holthouse.)

Amongst the causes of epilepsy mentioned by various writers, extreme sexual excesses are considered as not the least important. They would appear to have much influence on the frequency of the fits, as is shown in the narrative of the following case, the notes of which were taken by Mr. H. Ponsonby Adair, house-surgeon to the hospital. There are cases on record in which castration has been resorted to as a means of relief. In one reported by J. P. Frank, the *aura epileptica* began in the testicle, and it is asserted that a permanent cure followed castration.

This operation is much practiced amongst the Eastern nations, for the sole purpose of depriving their slaves of manhood; and Mr. Curling informs us, in his work on the "Diseases of the Testis," that in Italy it was once frequently performed on account of its effects on the vocal organs.

Eli B——, aged forty-four, widower, native of the United States, bookseller, was admitted into Luke ward in the above hospital, on the 4th of January, under the care of Mr. Holthouse, in order to have the operation of castration performed for the cure of epilepsy.

The patient is one of fourteen children, of whom eleven are living and healthy; his father is alive, aged eighty four, and his mother died at eighty. There is no insanity in the family, nor is any member of it afflicted with epilepsy. He was a healthy child till he was ten years of age, when he commenced to practice masturbation, and soon after had an epileptic fit, in which he bit his tongue. This was followed by severe pain in the head, and incapacity for exertion next day.—The fits recurred every three or four weeks. They came on suddenly without any premonitory symptoms. During the first two years he took "skull-cap tea," without effect; his diet was also regulated. He still continued to practice self-abuse, and did not finally relinquish it till he was twenty-two, about the time when he began to take nitrate of silver. For two years he tried homœopathy, the fits increasing in severity. He was at school up to the age of fifteen, when he tried a sea-voyage, but without benefit. Having returned, he sailed for South America, where he remained two years, the fits being as frequent as before. While at New York he contracted gonorrhœa, having been accustomed to frequent sexual intercourse from the age of sixteen, in addition to the habit of self-abuse. He remained in New York for a few months, trying various remedies, amongst them sulphate of zinc, but without relief. He went again to the south for a few months, and upon his return he placed himself under the care of Dr. Kissam, who prescribed nitrate of silver, in doses of one-eighth of a grain, three times daily, and in two months it was increased to half a grain. Very soon after he began to take this remedy the severity and frequency of the fits began to decrease, and he was so convinced of its efficacy, that he continued its use for about eight months, against the advice of Dr. Kissam, who feared it might affect his skin, which, indeed, it did to some extent, giving it a blue tint. At the end of this time, the fits left him for a period of two years, having gradually decreased in frequency under the use of the nitrate of silver. From the time of his contracting gonorrhœa till his marriage, he abstained altogether from sexual intercourse and the habit of self-abuse, so that during the whole time that he was taking the nitrate of silver he had no extraneous sensual excitement; yet during this period he says that he was constantly troubled with nocturnal erections, and frequent seminal emissions. Being now twenty-four years of age, he again became addicted to sexual excesses. He left his wife and his business for several months, and traveled; the fits, however, recurred every three or four weeks, and were very severe. On his return his wife died, and he remained a widower six years, abstaining altogether from sexual excesses, although frequently troubled with erections.—During the six years he broke his arm, several fingers, and his leg twice, whilst in the fits. At the age of thirty he married a second time, the fits having increased in number and severity. He was often compelled to send his wife into the country for a day or two, in order to avoid sexual excitement. The fits now recurred daily. His wife died a year after marriage. After this he again abstained from sexual excesses. Dr. Horace Green, of New York, now cauterized his larynx daily with nitrate of silver, and at the end of three or four months he would be free from fits for nineteen days; when they did recur, they were so slight that he scarcely lost consciousness, and did not fall down. This plan of treatment was pursued for two or three years, at the end of which time he became attached to another young woman, which revived all his old amatory feelings, and the fits began to increase in frequency, recurring at intervals of fourteen days, when they would continue daily for a week, and then cease for fourteen days more. Galvanism was now tried with some slight beneficial effect.—

Next arsenic, in the form of Fowler's solution, which he continued till the fits recurred daily, and he became so prostrate that he was confined to his bed. For a long time he took iron to neutralize the effects of the arsenic, but for months he was compelled to walk on crutches.—He came to England two years ago to have tracheotomy performed by Dr. Marshall Hall, who had advised it when he saw the man in America. Dr. Hall died soon after the man's arrival, and he went to Paris, and was under the care of M. Nélaton. Afterwards he placed himself under M. Trousseau, who gave him belladonna, which affected his vision but not his fits. Dr. de Lassiauve next treated him with camphor for four months without effect. He returned to England, and was under Mr. Simon, at St. Thomas's Hospital, in order to have castration performed, in which he had great faith, for he attributed his fits chiefly to sexual excitement, which still troubled him much; but his wish was not acceded to. He took bromide of potassium without any benefit, and then the nitrate of silver for two or three months, in half-grain doses three times a day. The skin became darker than before, and the fits recurred daily. He next went to Germany, and was there sounded for a stone in the bladder on account of frequent micturition, which he has had since infancy. No calculus was present. He was an inmate of the hospitals of Vienna, Prague, and Dresden. He left the latter in October, 1858, and was admitted into the Westminster Hospital, under Dr. Radcliffe, on the 30th of the month, and remained in two months, during which period he took quinine and iron, and camphor, but without avail. Since his second wife's death he has entirely abstained from sexual intercourse, though he has been constantly troubled with nocturnal erections, and occasional seminal emissions, and these continued up to the time when he came under the care of Mr. Holthouse, to whom he applied to perform castration, which after much deliberation he consented to do; and it was performed upon both testicles on the 4th of January, 1859, under the influence of chloroform. Two or three hours afterwards there was considerable hæmorrhage, which was checked by the application of cold. He had one fit during the hæmorrhage. His face has a bluish-slate tinge, which pervades the body, but the color is darkest on the face. His fits are of the rotary kind, preceded by a sudden scream, and lasting not more than a minute, and when over, he is quite himself again. In the fit which he had while in bed after the operation, he did not scream. but merely struggled violently.

Jan. 5th.—He had another fit this morning.

6th.—The fit recurred early this morning.

7th.—At four this morning another fit occurred. He says that after his second marriage the fits frequently followed immediately on the act of connexion.

8th.—Has had no fit at all to-day.

9th.—Had a very slight attack this morning, scarcely more than a giddiness for a minute. Altogether, since the operation, the fits have been exceedingly mild.—*London Lancet.*

